# The Mitchillean Theory Defended

**Published:** 1800-07

**Authors:** Samuel Lathom Mitchill


### *The Mitchillean Theory defended.*

[Extracted from a Letter of Profeffor MITCHILL, of New York, to Mr. PERKINS, of Leicefter Square, Proprietor of the Metallic Tractors. Dated Columbia College, April 3, 1800.]

I Think the *nitric* acid is fufficiently proved to be a mixture of *three* acids, in the pureft form in which it is obtained by chemifts.

Firft, There is a quantity of fea-falt mingled with the pureft falt petre, of which it is rarely or never divefted. This affords muriatic acid, which comes over firft in the diftillation, and mingles with the product in the receiver. The exiftence of this acid is proved by adding nitrate of filver, which is immediately rendered turbid, and precipitated in a white mucilaginous fediment.

Secondly, A portion of the fulphuric acid employed to decompofe the nitre, is volatalized by the heat, and rifes in vapour too; and thus a portion of fulphureous acid is blended with the diftilled fpirit in the receiver. This is proved by adding nitrat or acetat of lead, which falls down in the form of the fulphat of lead. Thefe precipitates are formed in all the forms and famples of nitrous acid that I ever faw.

Thirdly, It being known that the fulphuric and feptic acids are are mixible with each other, it muſt often happen that they combine *before* the mixture meets with potaſs to neutralize it.

Fourthly, As it is equally well eſtabliſhed, that ſeptic and muriatic acids will enter into union, it follows that theſe two, *very commonly indeed*, are blended together, *before* they are connected with the fixed vegetable alkali. It is therefore ordinarily impoſſible to procure one drop of pure and naked ſeptic acid, by any decompoſition of nitre that can be inſtituted. The acid obtained is always an *aqua regia*, or ſome ſuch thing; and the aſſertion that *aqua fortis* diſſolves ſilver, and *aqua regia* gold, is not correct. Both theſe acids are modifications of *aqua regia*; or, in other words, mixtures of various things with the original acid of putrefaction.

Fifthly, Such being the conſtitution of nitrous acid, the *nitric acid* muſt be equally a mixture or farrago of things; and thus, of courſe, is nitrous air, nitrous acid vapour, nitrous oxyd, and every thing of the ſort, procured by art, or by the proceſſes in the laboratory. I wiſh the gentlemen who preſcribe and publiſh ſo much about theſe ſubſtances, would conſider a little more attentively the compoſition of the things they employ; we ſhould then have leſs contradiction and obſcurity on the ſubject.

Sixthly, You may rely on it, therefore, that the *analyſis* of *nitrous acid* into *azote* and *oxygen*, is not correct—*There never has been ſuch a pure ſpecimen of nitrous acid in this world*, obtained by diſtillation from ſalt petre. I wiſh that the true compoſition of theſe forms and mixtures of ſeptic acid might be properly enquired into and underſtood.

Your's, with the higheſt conſideration and regard,

SAMUEL LATHOM MITCHILL.

